# Land-use change and greenhouse gas emissions from corn and cellulosic ethanol

**DOI:** 10.1186/1754-6834-6-51

**Published:** 2013-04-10

**Authors:** Jennifer B Dunn, Steffen Mueller, Ho-young Kwon, Michael Q Wang

**Affiliations:** 1 Systems Assessment Group, Argonne National Laboratory, 9700 South Cass Avenue, Argonne, IL 60439, USA; 2 Energy Resources Center, University of Illinois at Chicago, 1309 South Halsted Street, MC 156, Chicago, IL 60607, USA; 3 Department of Natural Resources and Environmental Sciences, University of Illinois at Urbana-Champaign, W-503 Turner Hall, MC-047, 1102 South Goodwin Avenue, Urbana, IL 61801, USA

**Keywords:** Ethanol, Land-use change, Life-cycle analysis, Soil carbon content

## Abstract

**Background:**

The greenhouse gas (GHG) emissions that may accompany land-use change (LUC) from increased biofuel feedstock production are a source of debate in the discussion of drawbacks and advantages of biofuels. Estimates of LUC GHG emissions focus mainly on corn ethanol and vary widely. Increasing the understanding of LUC GHG impacts associated with both corn and cellulosic ethanol will inform the on-going debate concerning their magnitudes and sources of variability.

**Results:**

In our study, we estimate LUC GHG emissions for ethanol from four feedstocks: corn, corn stover, switchgrass, and miscanthus. We use new computable general equilibrium (CGE) results for worldwide LUC. U.S. domestic carbon emission factors are from state-level modelling with a surrogate CENTURY model and U.S. Forest Service data. This paper investigates the effect of several key domestic lands carbon content modelling parameters on LUC GHG emissions. International carbon emission factors are from the Woods Hole Research Center. LUC GHG emissions are calculated from these LUCs and carbon content data with Argonne National Laboratory’s Carbon Calculator for Land Use Change from Biofuels Production (CCLUB) model. Our results indicate that miscanthus and corn ethanol have the lowest (−10 g CO_2_e/MJ) and highest (7.6 g CO_2_e/MJ) LUC GHG emissions under base case modelling assumptions. The results for corn ethanol are lower than corresponding results from previous studies. Switchgrass ethanol base case results (2.8 g CO_2_e/MJ) were the most influenced by assumptions regarding converted forestlands and the fate of carbon in harvested wood products. They are greater than miscanthus LUC GHG emissions because switchgrass is a lower-yielding crop. Finally, LUC GHG emissions for corn stover are essentially negligible and insensitive to changes in model assumptions.

**Conclusions:**

This research provides new insight into the influence of key carbon content modelling variables on LUC GHG emissions associated with the four bioethanol pathways we examined. Our results indicate that LUC GHG emissions may have a smaller contribution to the overall biofuel life cycle than previously thought. Additionally, they highlight the need for future advances in LUC GHG emissions estimation including improvements to CGE models and aboveground and belowground carbon content data.

## Background

Biofuels are often considered to be among the technologies that can reduce the greenhouse gas (GHG) impacts of the transportation sector. Yet the changes in land use that could accompany the production of biofuel feedstocks and the subsequent environmental impacts, including GHG emissions, are a potential disadvantage of biofuels. Land-use change (LUC) occurs when land is converted to biofuel feedstock production from other uses or states, including non-feedstock agricultural lands, forests, and grasslands. This type of LUC is sometimes called direct LUC. The resulting change in crop production levels (e.g., an increase in corn production may cause a decrease in soybean production) and exports may shift land uses domestically and abroad through economic linkages. This latter type of LUC is called indirect LUC and can be estimated through the use of economic models.

A change in land use causes a change in carbon stocks aboveground and belowground. As a result, a given LUC scenario may emit or sequester carbon. When an LUC scenario results in a net release of carbon to the atmosphere, it is debated if biofuels result in GHG reductions at all [1,2]. Of particular concern is the conversion of forests [3,4], an inherently carbon-rich land cover that in some cases may be a carbon sink. Their conversion to biofuel feedstock production land could incur a significant carbon penalty [5].

The estimation of LUC and the resulting GHG emissions is accomplished through the marriage of LUC data with aboveground carbon and soil organic carbon (SOC) data for each of the land types affected. The amounts and types of land converted as a result of increased biofuel production can be estimated with an agricultural-economic model, for example, a computable general equilibrium (CGE) model; several recent reports [6,7] provide an overview of CGE models and their application to estimating LUC associated with biofuel production. It is also necessary to know the aboveground and belowground carbon content of the land in its original state and in its future state as feedstock production land. Aboveground carbon content information is provided by databases that are often built on satellite data [8], while SOC content can be modelled with tools such as CENTURY [9].

LUC GHG emissions from biofuel production are typically placed in the context of a biofuel life cycle analysis (LCA), which estimates the GHG emissions of a biofuel on a farm-to-wheels basis [10]. Regulatory bodies, including the U.S. Environmental Protection Agency (EPA), the California Air Resources Board (CARB), and the European Union [11-13], use LCA to evaluate the GHG impacts of biofuels.

When LUC GHG emissions are examined in the context of a biofuel’s life cycle, they can be substantive. For example, EPA estimated that LUC GHG emissions were 38% of total life cycle GHG emissions for corn ethanol produced in a natural gas-powered dry mill with dry distillers grains solubles (DGS) as a co-product [11]. LUC GHG emissions are also highly uncertain [14] due to large uncertainties in CGE modelling, aboveground carbon data, and SOC content data [15].

As one of the most prevalent biofuels, corn ethanol has been the subject of most biofuel LUC research [14,16]. Few studies have considered LUC GHG emissions from cellulosic ethanol production. Hill et al. [17] estimated domestic LUC GHG emissions for the production of 3.8 billion litres of ethanol based on conversion of land formerly in the Conservation Reserve Program (CRP) to production of corn, corn stover, switchgrass, prairie grass, and miscanthus. The resulting LUC GHG emissions for corn were between 27 and 35 g CO_2_e/MJ. These emissions were 0.5 and 0.2 g CO_2_e/MJ for switchgrass and miscanthus, respectively. Corn stover was assumed to have no LUC GHG emissions associated with its production. Scown et al. [18] considered a number of domestic U.S. scenarios for the production of 39.7 billion liters/year of ethanol from miscanthus, allowing only cropland or CRP lands to be converted to miscanthus production. These authors modelled productivity of miscanthus with Miscanmod at the county level. A model proposed by Matthews and Grogan [19] was used to estimate the SOC content of converted land. SOC changes were aggregated to the county level from a 90-meter resolution. In their calculation of LUC GHG emissions, Scown et al. [18] did not consider the impact of land management history on SOC content. Their study concluded that on net 3.4 to 16 g CO_2_e/MJ would be sequestered as a result of SOC changes. Separately, Davis et al. [20] considered the conversion of 30% of domestic (U.S.) land currently in corn production to miscanthus or switchgrass (fertilized or unfertilized) production. They used DAYCENT to simulate regional miscanthus and switchgrass cultivation in the central U.S. and identified lower GHG fluxes from cultivation when either crop was grown in place of corn. The reductions after 10 years (1.9% for switchgrass with fertilization and 19% for miscanthus) came from both reduction in fertilizer-derived N_2_O emissions and increased carbon sequestration. Similarly, Qin et al. [21] showed that SOC content increases by 50 and 80% when land is converted from corn cultivation to switchgrass and miscanthus, respectively. EPA has estimated LUC GHG emissions for cellulosic ethanol derived from corn stover (−10 g CO_2_e/MJ) and switchgrass (12 g CO_2_e/MJ) [11]. CARB has examined forest residue and farmed trees as feedstocks for cellulosic ethanol [22,23]. The agency developed preliminary LUC GHG estimates for the latter feedstock, which is not examined in our current study.

The above literature summary highlights two limitations of previous studies of LUC GHG emissions associated with cellulosic ethanol production. First, application of worldwide CGE modelling to LUC GHG calculations for cellulosic ethanol has been limited to EPA and CARB analyses for switchgrass and corn stover. Second, SOC emission factors have either been developed for very specific lands (e.g., CRP or agricultural lands) or at the national or regional scale for other land types, as in the CARB and EPA analyses. In our study, we sought to address these two limitations of the current literature.

First, we used worldwide LUC results for four biofuel production scenarios (Table 1) as modelled with Purdue University’s Global Trade Analysis Project (GTAP) CGE model [24]. The modelling considered domestic U.S. production of ethanol from four feedstocks: corn, corn ethanol, switchgrass, and miscanthus. Second, we applied finer-level SOC emission factors (EF) than have been used in previous analyses for all land categories, including forests. We developed a modelling framework to estimate these EFs at the state-level by utilizing remote sensing data, national statistics databases, and a surrogate model for CENTURY’s soil organic C dynamics submodel (SCSOC) [25]. Details of the development of these EFs, which account for both aboveground and belowground carbon content changes, are provided in the Methods section and in a separate publication [26] as is the handling of international carbon EFs [27]. The LUC and carbon EF data were compiled in Argonne National Laboratory’s Carbon Calculator for Land Use Change from Biofuels Production (CCLUB) model to enable calculation of LUC GHG emissions [28]. CCLUB is a module of Argonne National Laboratory’s Greenhouse Gases, Regulated Emissions, and Energy use in Transportation (GREET™) model which was used to analyse LUC GHG emissions in the context of overall bioethanol life-cycle GHG emissions. GREET covers bioethanol production pathways extensively and is used by Argonne and other researchers to examine GHG emissions from transportation fuels and vehicle technologies [28].

**Table 1:** **GTAP modelling scenarios**[24]

**Scenario**	**Scenario description**	**Increase in Ethanol (BL)**
1	An increase in corn ethanol production from its 2004 level of 13 billion litres (BL) to 57 BL	45
2	An increase of ethanol from corn stover by 35 BL, in addition to 57 BL corn ethanol	35
3	An increase of ethanol from miscanthus by 27 BL, in addition to 57 BL corn ethanol	27
4	An increase of ethanol from switchgrass by 27 BL, in addition to 57 BL corn ethanol	27

In this paper, we estimate LUC GHG emissions associated with ethanol produced from four feedstocks (corn, corn stover, switchgrass, miscanthus). A sensitivity analysis is conducted to investigate the influence of key carbon content modelling assumptions on results. Addressing CGE model assumptions and their impact on LUC GHG emissions is outside the scope of this paper.

## Results and discussion

**Figure 1:**
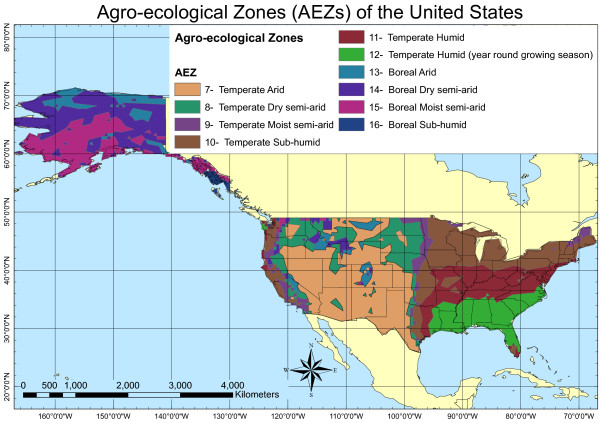
Distribution of AEZs in the United States.

In the following subsections, we describe LUC, domestic U.S. aboveground carbon, and domestic U.S. SOC modelling results. Next, we provide a full discussion of LUC GHG emissions results and place them in the context of life-cycle GHG emissions for each biofuel. The discussion is based on an agro-ecological zone (AEZ) level although SOC EFs for domestic U.S. lands were determined at a state level [27]. Figure 1 provides the distribution of AEZs in the United States for reference.

### Land-use change

**Figure 2:**
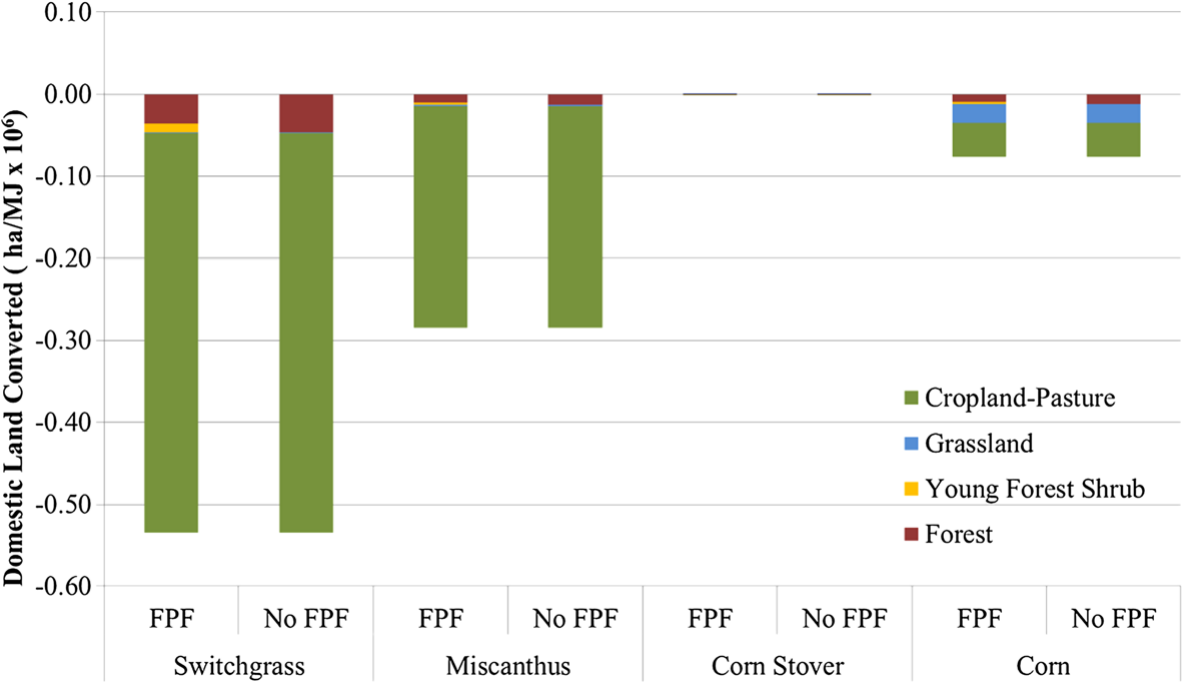
**Domestic LUC for switchgrass, miscanthus, corn stover, and corn ethanol.** Legend: Negative values indicate a decrease in land area.

In this paper, we divide LUC into domestic and international LUC for clarity and simplicity because it is not possible to distinguish between direct and indirect LUC in GTAP results, which are calculated at an AEZ level in the United States and a country/regional level abroad. As described above, types and amounts of converted lands were modelled with GTAP using four scenarios (Table 1) designed to follow the arc of Renewable Fuel Standard (RFS2) implementation. First, corn ethanol production expands until the RFS2 limit of 57 billion litres (BL) is met. Subsequently, cellulosic ethanol feedstocks will be produced on lands that corn does not already occupy. Results for each feedstock are presented in Figure 2. We developed and applied a forest proration factor (FPF) to adjust total domestic forest area converted for production of these feedstocks [27]. We took this approach to align forest land areas in the GTAP land database, the National Land Cover Dataset, and the U.S. Forest Service Forest Inventory Data. This step was necessary for consistency in the analysis because we used the latter to develop emission factors for aboveground and belowground carbon in addition to values for foregone sequestration. GTAP contains significantly more forested land than either of the other two data sources. When applying the FPF reduces the amount of forest converted, the difference is made up with land covered by young, thin trees. In Figure 2, this land type is called Young Forest-Shrub (YFS). The forest emissions factor for YFS is based on the relative height of forest stands in each state compared to shrubland. The relative tree heights for each state were derived from Pflugmacher et al. [29] and Buis [30]. When we apply the FPF, between 20 and 22% of converted land shifts from forests to YFS for all feedstocks.

In the case of corn ethanol (Scenario 1 in Table 1), most of the land converted in the U.S. is cropland-pasture along with some domestic forest (Figure 2). Modelling results indicated that AEZ 10 (temperate sub-humid) is most affected by expansion of corn agriculture. Of the cellulosic feedstocks, corn stover has the lowest impact on domestic land use. Although this feedstock has the lowest productivity (Table 2), this result is unsurprising because stover is modelled as a “waste” product of corn production (as opposed to a co-product). Stover harvesting may not fundamentally change corn farming and should not result in significant LUC. Additionally, the greater amount of land converted for switchgrass ethanol production as compared to miscanthus ethanol production in the U.S. can be explained by crop yield, which can be nearly two times higher for miscanthus [31,32]. For both switchgrass and miscanthus ethanol, the majority of the land converted is in AEZ 7 (temperate arid) and is cropland-pasture. Nonetheless, the amount of forest converted for switchgrass is striking.

**Table 2:** Feedstock productivity

**Feedstock**	**Crop yield (dry metric ton/ha)**	**Ethanol productivity (L/ha)**
Corn	7.9^a^	4,250 L/ha^b^
Miscanthus	24^c^	6,190 L/ha^d^
Switchgrass	12^c^	3,200 L/ha^d^
Corn stover	4.1^c^	1,070 L/ha^d^

^a^ Yield calculated from [28] and 20% moisture content at harvest.

^b^ 344 L/dry metric ton [28].

^c^ From [24].

^d^ Assuming an ethanol yield of 317 L/dry metric ton [24].

**Figure 3:**
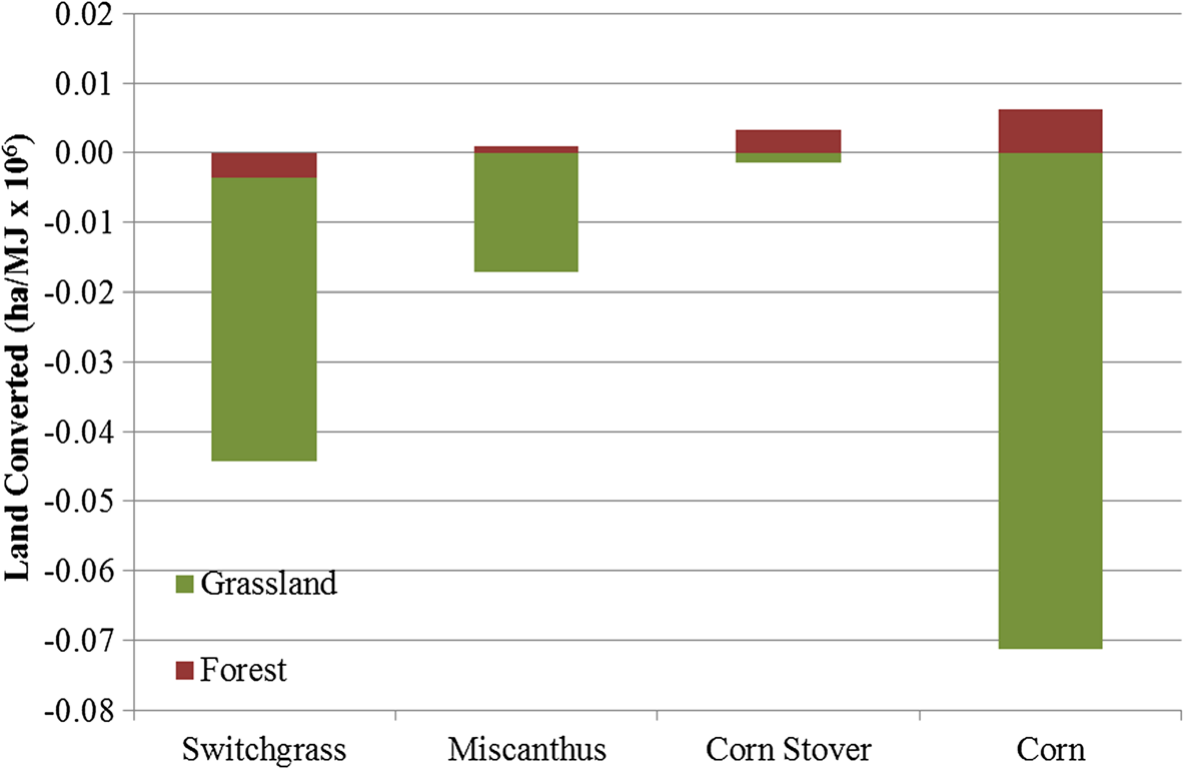
**International LUC for switchgrass, miscanthus, corn stover, and corn ethanol.** Legend: Negative and positive values indicate a decrease and increase, respectively, in land area.

Figure 3 displays international LUC that occurs from production of corn and cellulosic ethanol. Internationally, corn causes more LUC than the other crops because, unlike the cellulosic crops, U.S. corn accounts for a large share of the international corn market and a reduction in U.S. corn exports caused by corn ethanol production increases corn production in other countries. Among cellulosic crops, switchgrass production causes the most land conversion and, as it does domestically, the highest amount of forest conversion. (Note that no FPF was applied for international forest conversions.) Switchgrass production consumes cropland-pasture land in the United States, possibly shifting agricultural production from these lands to other countries. For both corn and corn stover feedstocks, some forest land is recovered internationally, the majority of which is in Russia. Table 3 shows domestic, international, and total LUC for each feedstock.

**Table 3:** **Total domestic and international LUC for each feedstock (ha/MJ × 10**^**6**^**)**

**Feedstock**	**Domestic**	**International**	**Total**
Switchgrass	−0.54	−0.04	−0.58
Miscanthus	−0.29	−0.02	−0.30
Corn stover	5.7 × 10^-4^	1.8 × 10^-3^	2.4 × 10^-3^
Corn	−0.08	−0.07	−0.14

### Soil organic carbon emission factors

**Figure 4:**
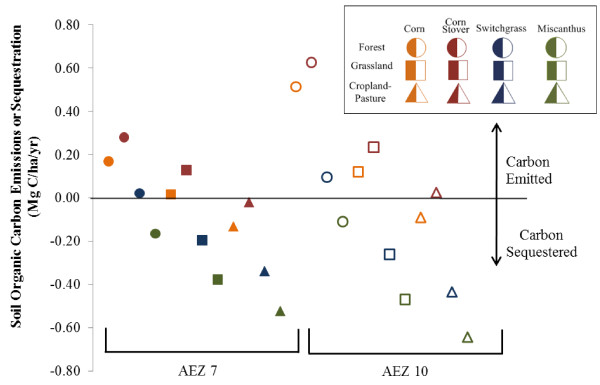
**Soil organic carbon content changes from domestic land-use transitions.** Legend: Solid and hollow markers denote transitions in AEZs 7 and 10, respectively. Forest, grassland, and cropland-pasture transitions are denoted by circles, squares, and triangles, respectively. Orange, red, green, and blue markers reflect transitions to corn, corn stover, miscanthus, and switchgrass production, respectively. These results were generated from surrogate CENTURY modelling runs with calibrated soil cultivation effect coefficients, feedstock yields that increase with time, and with erosion effects.

The development of the SOC EFs used in CCLUB is summarized in the Methods section, a detailed discussion can be found in an earlier publication [26]. Here, we discuss trends in these EFs and the implications for LUC GHG emissions. The variation in SOC EFs with location, a result of soil type and climate differences, is an important feature of this analysis. Although state-level EFs were calculated for each land transition and biofuel scenario, in CCLUB these EFs are rolled up to an AEZ level to match AEZ-level GTAP results. In Figure 4, we present the variation of SOC EFs for two AEZs (7 and 10) that GTAP predicts will experience the largest amount of LUC by feedstock and land conversion type. These results were generated from modelling runs with calibrated surrogate CENTURY soil cultivation effect coefficients, feedstock yields that increase with time, and with erosion effects (surrogate CENTURY case sd in Table 4). (We discuss the influence of surrogate CENTURY modelling choices on LUC GHG emissions in the next section.) Clearly, conversion of forest to produce corn or corn-corn stover results in the greatest amount of carbon emissions. Forest conversion to miscanthus production, however, may not incur a carbon penalty. Carbon sequestration occurs when grassland or cropland-pasture is converted to switchgrass or miscanthus production, which is consistent with other studies [20,21]. The data in Figure 4 consistently show that, of the land use transitions we considered, conversions to miscanthus maximize carbon sequestration. This result is consistent with miscanthus growth generating more aboveground and belowground biomass [26]. The SOC emission factors vary slightly between AEZs 7 and 10 with the exception of forest land converted to corn production. Converting forest to corn or corn stover production in AEZ 10 will produce greater carbon emissions than this transition in AEZ 7.

**Table 4:** Surrogate CENTURY scenarios in CCLUB

**CCLUB case**	**Soil cultivation effect coeffecient**		**Crop yield**		**Erosion**	
	**CENTURY default**	**Calibrated**	**Increase**	**No increase**	**Erosion**	**No erosion**
sa	X			X		X
sb	X		X		X	
sc	X			X	X	
sd		X	X		X	
se		X		X	X	

**Figure 5:**
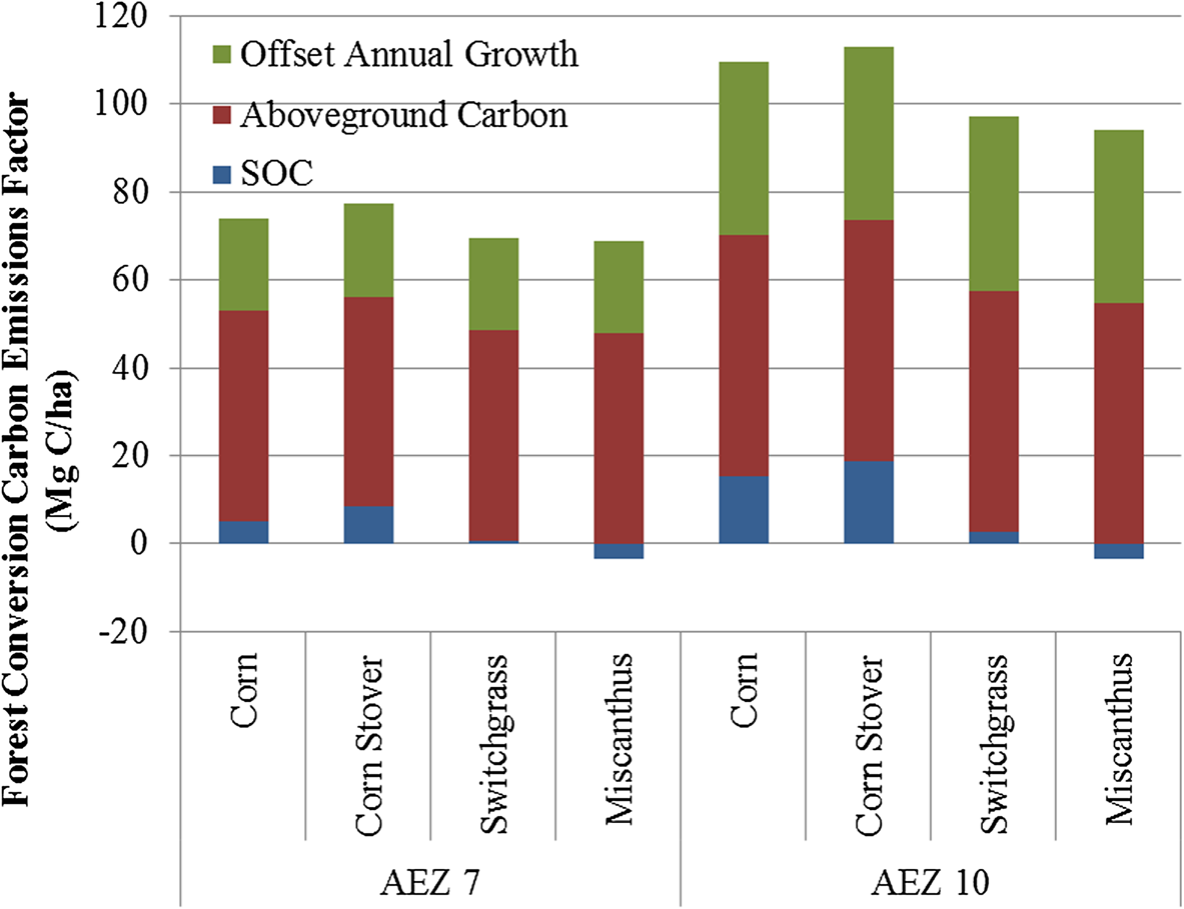
**Forest carbon emission factor for four feedstocks in AEZs 7 and 10.** Legend: SOC values were calculated with modelling option “sd” in Table 4.

In estimating GHG emissions from the conversion of forests to biofuel feedstock production lands, we consider two sources of aboveground carbon: carbon contained in aboveground biomass that is cleared and the loss of carbon sequestration that would have occurred if the forest had continued to grow. See Mueller et al. [27] for a full discussion of how these factors were developed. Figure 5 breaks down the total carbon emissions factor applied to converted forest land for each feedstock in AEZs 7 and 10. The largest contributor to these emission factors is aboveground carbon. Both aboveground carbon and carbon sequestered during annual growth are greater in AEZ 10 than in AEZ 7. As expected based on Figure 4, conversion of forest to corn production with stover harvest transitions incur the greatest carbon penalty whereas transition to miscanthus production results in the lowest amount of GHG emissions.

### LUC GHG emissions

CCLUB is populated with carbon EFs generated from surrogate CENTURY modelling under four scenarios outlined in Table 4. The scenarios differ in their treatment of three key parameters: soil erosion, crop yield, and the soil cultivation effect coefficient. The latter was either left at default values or calibrated to real-world data. Additionally, EFs were also produced under different land management practices (conventional till, reduced till, no-till) for corn and corn stover feedstocks. We selected scenario “sd” in Table 4 as the base case for this study. For corn with and without stover harvest, the land management practice of conventional till is the base case setting.

#### Base case LUC GHG results

**Figure 6:**
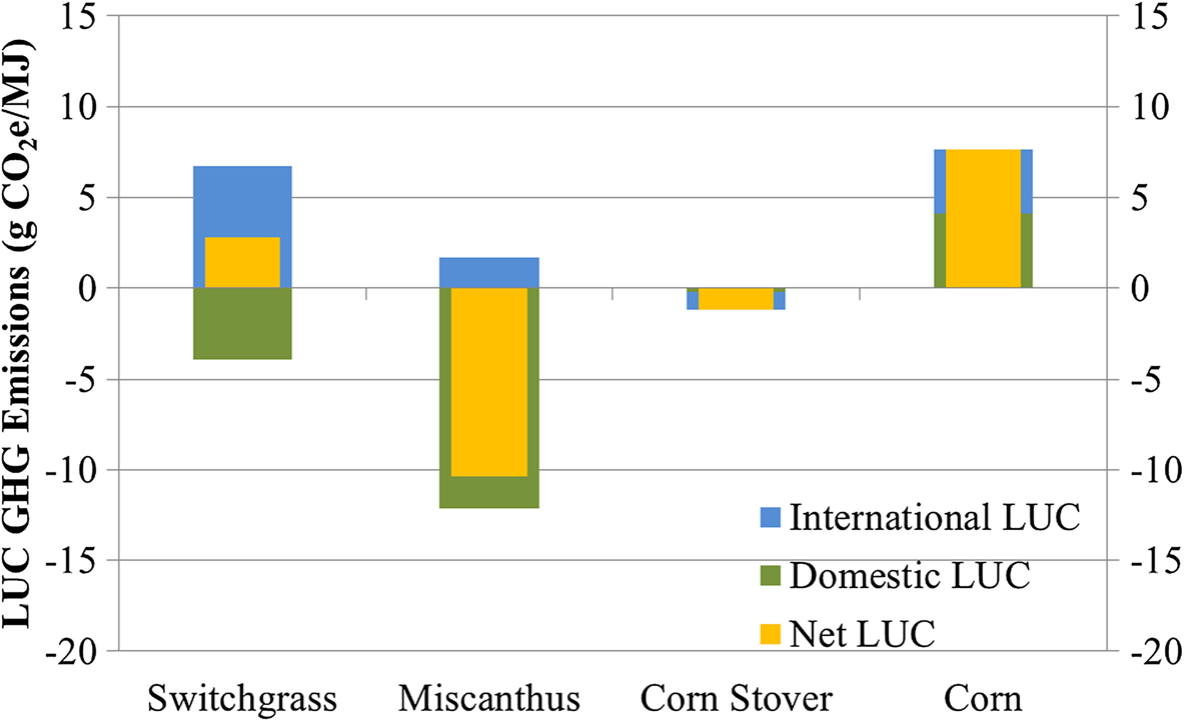
**Base case LUC GHG emissions (g CO**_**2**_**e/MJ) for switchgrass, miscanthus, corn stover, and corn ethanol.** Legend: Domestic LUC GHG emissions were calculated with modelling option “sd” in Table 4, adopting the FPF, and assuming sequestration of 42% of aboveground live and dead tree carbon in HWP.

**Figure 7:**
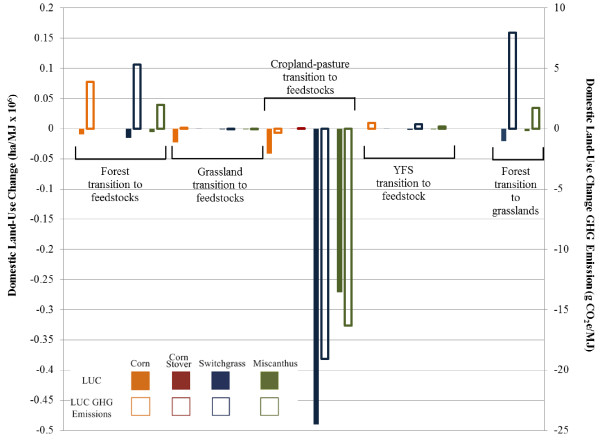
**LUC (ha/MJ** × **10**^**6**^**) and LUC GHG emissions (g CO**_**2**_**e/MJ) from selected land conversions.** Legend: Solid and hollow bars represent LUC amounts and LUC GHG emissions, respectively. Orange, red, blue, and green bars indicate feedstocks of corn, corn stover, switchgrass, and miscanthus, respectively. Results reflect base case modelling conditions.

Figure 6 contains the base case LUC GHG emissions results for the four bioethanol production scenarios in Table 1. Figure 7 pairs domestic U.S. LUC for each feedstock with the resulting base case domestic GHG emissions or sequestration. In the U.S., the miscanthus ethanol scenario causes significant SOC increases in the large amount of cropland-pastureland converted for feedstock growth. International LUC GHG emissions associated with this scenario are positive, but minimal. Miscanthus ethanol then exhibits net GHG sequestration from LUC. In the case of switchgrass ethanol, international LUC GHG emissions are significant. As described earlier, switchgrass production converts large areas of domestic cropland-pasture land, triggering conversion of lands abroad, including forests, to agriculture. In the United States, GHG emissions from forest-to-switchgrass conversion cut into gains in soil carbon from conversion of cropland-pasture lands to switchgrass production (Figure 7). The switchgrass ethanol scenario therefore on net emits GHGs as a result of LUC. Less land is converted for corn ethanol production than for switchgrass, yet LUC GHG emissions for corn ethanol exceed those for all cellulosic crops. LUC GHG emissions for corn ethanol are not offset by sequestration elsewhere (Figure 7) because corn reduces or only minimally enhances SOC (Figure 4). The results when corn stover is the ethanol feedstock show a small amount of carbon is sequestered. LUC modelling in this case predicts slight domestic gains in both YFS and forest lands and an increase in international forest lands, which sequester enough carbon to offset the carbon emitted from cropland-pasture conversion. For the most part, however, LUC GHG impacts of corn stover ethanol production can be considered negligible.

#### Effect of key surrogate CENTURY model parameters

**Figure 8:**
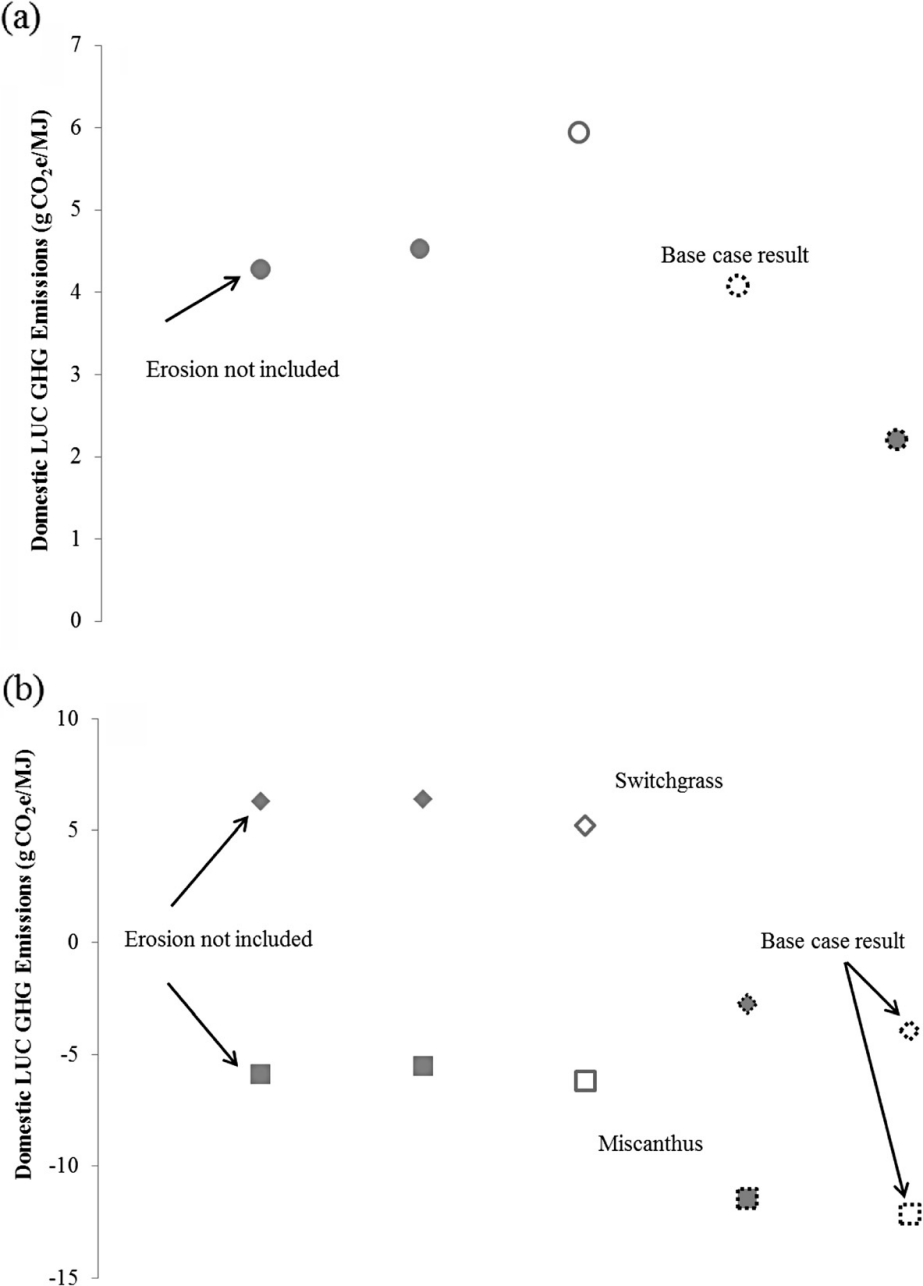
**Surrogate CENTURY parameters’ impact on domestic ethanol LUC GHG emissions for (a) conventionally-tilled corn (b) switchgrass and miscanthus.** Legend: Solid and hollow shapes indicated surrogate CENTURY modelling with default and calibrated soil cultivation effect coefficients, respectively. Shapes with solid and dashed outlines represent surrogate CENTURY runs with constant and increasing yields, respectively. Diamond markers represent switchgrass results; square markers represent miscanthus results. All results except those indicated include erosion effects. In all cases, HWP sequesters 42% of aboveground live and dead tree carbon and the FPF is applied.

Next, we consider how three surrogate CENTURY modelling choices affect these base case domestic LUC GHG emission results for corn ethanol (with conventional till) (Figure 8a) and for miscanthus and switchgrass ethanol (Figure 8b). The first modelling choice is whether to use a default or calibrated soil cultivation coefficient. Called *clteff*, this coefficient represents acceleration in soil carbon decay as a result of cultivation and fertilization under corn-based agriculture. Because it is used to establish the baseline amount of SOC in cropland before switchgrass or miscanthus production begins, it influences results for these feedstocks. Its calibrated value is larger than the default value [25]. Applying the calibrated soil cultivation effect coefficient therefore increases emissions from corn production. On the other hand, emissions decrease slightly from switchgrass and miscanthus production because when more SOC decay occurs prior to establishment of the feedstocks (calibrated *clteff*), conversion of cropland to produce them yields larger SOC increases. The second modelling choice is whether to assume crop yields are static or increasing. To investigate the influence of assuming crop yields increase, a 1% annual increase in yield for miscanthus and switchgrass was assumed [33]. Corn yield increases were based on historical data [25]. Crop yield increases translate into the production of more belowground carbon, some of which would be incorporated into SOC. Logically, then, assuming crop yields increase with time causes LUC GHG emissions to decline regardless of feedstock. Finally, the impact of soil erosion can be included. Erosion would be expected to decrease SOC, but Figure 8 illustrates that including its impact has a limited effect on domestic LUC GHG emissions.

#### Effect of key CCLUB model parameters

**Figure 9:**
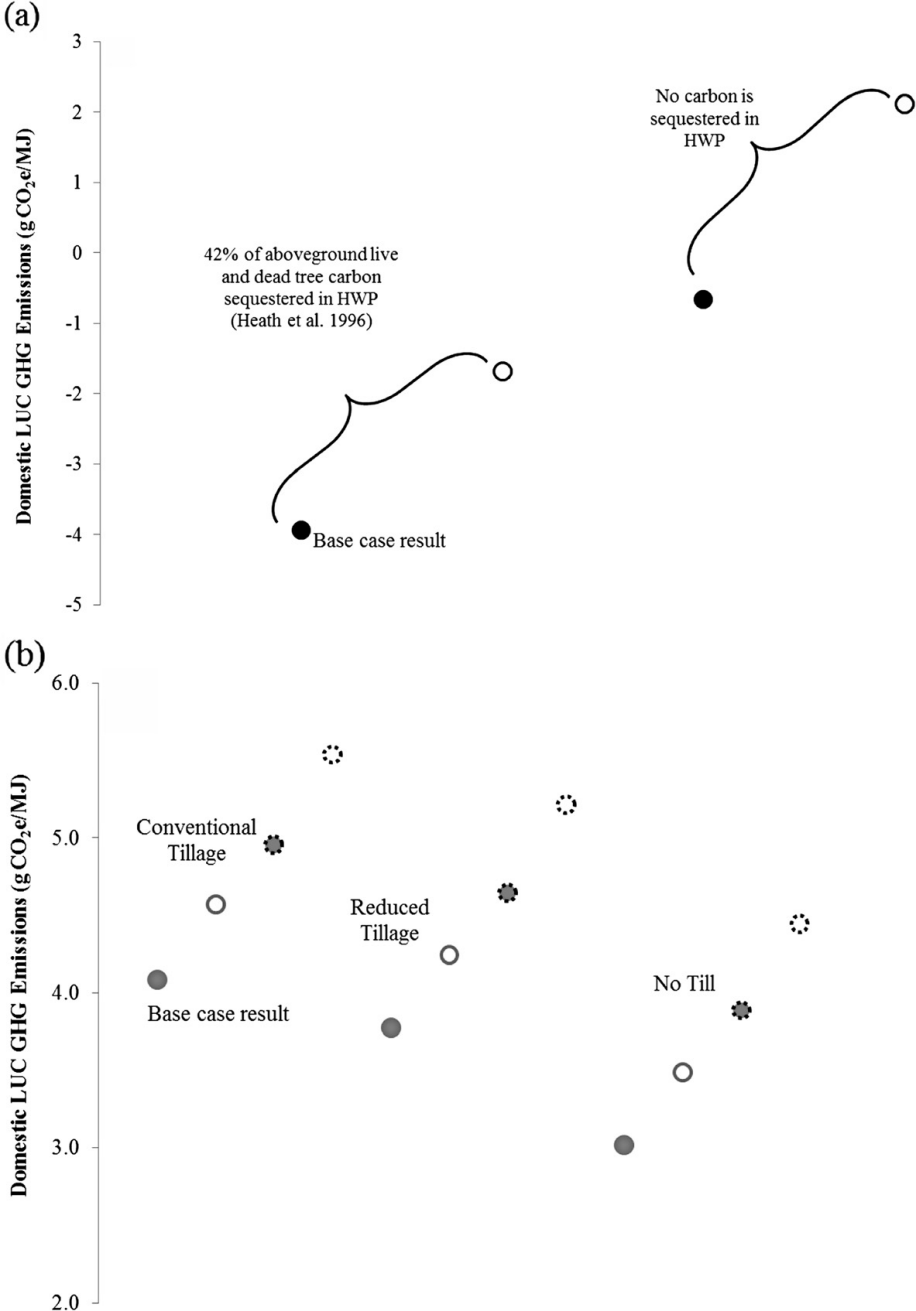
**HWP and FPF impact on domestic ethanol LUC GHG emissions for (a) switchgrass and (b) corn.** Legend: In Figures 9a and 9b, solid circles represents results calculated with the FPF applied. Hollow circles represent results using default GTAP results for area of converted forests. In Figure 9b, solid bordered circles represent results that account for some carbon sequestration in HWP. Circles with dashed borders are used for results that assume no carbon is sequestered in HWP.

In addition to containing EFs from surrogate CENTURY modelling under the scenarios in Table 4, CCLUB allows users to explore the effect of two other variables, the fate of carbon in harvested wood products (HWP) (e.g., lumber for buildings) and amount of forested land area in the U.S. (which can be determined with or without the FPF). In the case of HWP, one CCLUB scenario assumes 42% of aboveground live and dead tree carbon is sequestered in HWP [34]. The alternative scenario is that all carbon in these products is emitted. Figures 9a and 9b examine the impact of HWP and FPF for switchgrass and corn ethanol, respectively. We examine switchgrass results because GTAP predicts its production converts the largest amount of forests. In Figure 9a, accounting for sequestration of carbon in HWP reduces LUC GHG emissions by between 3 and 4 g CO_2_e/MJ when the FPF assumption is held constant. For a given HWP assumption, applying the FPF decreases GHG emissions by between 2 and 3 g CO_2_e/MJ.

In the case of corn ethanol (Figure 9b), applying the FPF decreases emissions by less than 1 g CO_2_e/MJ when the type of tillage and the HWP assumption are held constant. Changing the HWP assumption under a constant tillage and FPF scenario decreases emissions by approximately 1 g CO_2_e/MJ. As expected, for a given HWP and FPF configuration, corn grown under a no-till land management practice emits less carbon because tillage activities do not disturb the soil and release carbon to the atmosphere.

#### Biofuel LUC GHG emissions in a life-cycle context

In Table 5, we provide the range of LUC GHG emissions results that can be obtained by varying the key surrogate CENTURY and CCLUB modelling parameters as described above. We also provide the range of life-cycle GHG emissions assuming the default GREET assumptions for each ethanol pathway [10]. Without the contribution of LUC GHG sequestration, the net life-cycle GHG emissions result for miscanthus ethanol would be positive. Scown et al. [18] reported slightly higher GHG sequestration (between −3 and −16 g CO_2_e/MJ) from miscanthus production, but limited their study to active cropland or CRP land. LUC GHG emissions could potentially contribute significantly to life-cycle GHG emissions (up to 19 g CO_2_e/MJ) for switchgrass ethanol. This fuel exhibits the largest sensitivity to changes in modelling parameters in LUC GHG emissions. The area of forest that is predicted to be converted to grow this feedstock makes switchgrass results more sensitive to assumptions about HWP and the FPF than results for the other feedstocks (Figure 9). Corn ethanol LUC results vary considerably, although the base case estimate (7.6 g CO_2_e/MJ) aligns well with a value in another recent report [35]. At most, LUC GHG emissions contribute 20% of life-cycle GHG emissions for corn ethanol. Regardless of the modelling scenario, corn stover ethanol LUC GHG emissions are essentially negligible.

**Table 5:** **Range of LUC GHG emissions (g CO**_2_**e/MJ)**^**a**^

	**Switchgrass**	**Miscanthus**	**Corn stover**	**Corn**
Minimum U.S. LUC GHG emissions	−3.9	−12	−0.24	1.2
Maximum U.S. LUC GHG emissions	13	−3.8	−0.19	7.4
International LUC GHG emissions	6.7	1.7	−0.97	3.5
LUC GHG emissions range	2.7 to 19	−10 to −2.1	−1.21	4.7 to 11
Lifecycle GHG emissions range^b^	10 to 26	−8.5 to −0.20	0.97 to 1.0	62 to 68

^a^ Values presented represent range of results generated at all combinations of surrogate CENTURY (Table 4) and CCLUB modelling parameter settings discussed.

^b^ Using default GREET parameters [10] and varying only LUC GHG emissions.

## Conclusions and future research

In this research, we have examined LUC GHG emissions of ethanol from four feedstocks: corn, corn stover, switchgrass, and miscanthus. Of the fuels examined, corn ethanol has the highest LUC GHG emissions. However, the estimate of LUC GHG emissions for this fuel has decreased substantially compared to earlier studies [1,2,11,12,36]. This evolution is due to improvements in CGE modelling such as modifications to the modelling of animal feed, yield responses to price increases, and representation of growth in both supply and demand [24].

Miscanthus ethanol shows the potential to sequester carbon over the course of its life cycle. This result is largely due to its high yield. Scown et al. [18] reached a similar conclusion, although they predict a higher amount of carbon sequestration from miscanthus production-induced LUC. On the other hand, switchgrass exhibits higher emissions than miscanthus because it is produced with a lower yield, necessitating more land, including carbon-rich forests, to be converted for its production. It is important to note that the contrast between switchgrass and miscanthus results is largely due to the difference in their yield. Similar differences may be observed between other high- and low-yield energy crops. LUC GHG emissions associated with corn stover were negligible. As the technology for corn stover’s conversion to biofuels and other uses matures, corn stover may evolve into a co-product of corn production rather than a waste product. In that case, future modelling efforts could allocate LUC GHG impacts between the two fuels.

The sensitivity of LUC GHG emissions to key modelling parameters that dictate carbon emissions from converted lands is highlighted from the range of possible results in Table 5, which are affected by belowground and aboveground carbon simulation assumptions and results. As discussed, we did not investigate the influence of key CGE parameters on emissions because we used only one set of GTAP results. The uncertainty associated with these models, including GTAP, is large and difficult to estimate, as Plevin et al. [14] discuss. Improvements to these models, including modelling scenarios in which multiple feedstocks are simultaneously produced, scenarios at higher resolution (state or county-level), and scenarios with dynamic crop yields will shed further light on biofuel-induced LUC and better inform estimates of subsequent GHG emissions.

Improvements to estimates of converted lands’ carbon content are also needed. First, SOC content data for soils worldwide is needed, as explained in Smith et al. [8], who provide a vision for developing these data and discuss key sources of uncertainty in their development. Soil organic matter models such as CENTURY would benefit from further calibration of default parameters, including the soil cultivation effect coefficient, with real-world data.

Additionally, it is important to include other factors that accompany LUC beyond soil carbon changes, as we have considered. For example, nitrogen fertilization rates will change, depending on the land use both on the site of feedstock production and at other, indirectly affected agricultural sites, affecting N_2_O emissions rates from the soil. The EPA has considered indirect effects like these [11]. Further, Georgescu et al. [37] examine the effects of stored soil water, which can have a regional cooling effect, as impacted by LUC. Additionally, land cover albedo will change with LUC [38]. Because the uncertainty that surrounds biofuel LUC impacts are a key barrier to what otherwise may be a technology that offers environmental and energy security benefits, these impacts certainly merit further study. It is important to realize, however, that the complexity inherent in modelling worldwide phenomena in the future that involve economic, biogeochemical, and biogeophysical effects will likely always lead to large uncertainties and will produce estimates of LUC GHG emissions that vary widely.

Despite the uncertainty and complexity associated with estimating LUC GHG emissions, the continued pursuit of improvement of these estimates will increase understanding of crop management practices that limit GHG emissions from SOC depletion, provide new data for policy formulation that limits LUC impacts through, for example, preventing conversion of carbon-rich lands (forests), and identify crops that minimize LUC GHG emissions when produced on a large scale as biofuel feedstocks.

## Methods

To conduct the modelling for this analysis, we used Argonne National Laboratory’s CCLUB and GREET models [28]. The GREET model is developed at Argonne National Laboratory and is widely used to examine GHG emissions of vehicle technologies and transportation fuels on a consistent basis. CCLUB combines land transition data from GTAP modelling [24] with carbon emission factors derived from several sources. Domestic SOC content data were developed with a surrogate model for CENTURY’s soil organic carbon submodel (SCSOC) [25,26]. In this modelling, we estimated the forward change in soil C concentration within the 0–30 cm depth and computed the associated EFs for the 2011 to 2040 period for croplands, grasslands or pasture/hay, croplands/conservation reserve, and forests that were suited to produce any of four possible biofuel feedstock systems (corn-corn, corn-corn with stover harvest, switchgrass, and miscanthus). This modelling accounted for prior land-use history in the U.S. dating to 1880. SOC modelling was conducted under a number of parameter settings to examine the effect of soil erosion, crop yield increases, and the calibration of values for a key coefficient that represents the soil cultivation effect. Surrogate CENTURY modelling scenarios are shown in Table 4. Additionally, the effect of three different land management (tillage) scenarios for corn and corn stover production were examined: conventional till, no till, and reduced till. Our modelling of conventional tillage assumes that 95% of surface residues are mixed with soils, whereas no-tillage scenarios assume a converse 5% mixing of surface soils.

International SOC emission factors were adopted from data from the Woods Hole Research Center. The data, available at the biome level, were authored by R. Houghton and provided to CARB and Purdue University to support land-use modelling. Tyner and co-authors [36] reproduced the data set. We incorporated aboveground carbon emissions impacts of forest conversion using data from the U.S. Department of Agriculture’s (USDA) Forest Service/National Council for Air and Stream Improvement, Inc. (NCASI) Carbon Online Estimator (COLE) [39]. Technical documentation for CCLUB is available [27]. GREET parameters for feedstock production and growth are provided in several reports [31,32,40]. Other bioethanol life cycle parameters are provided in Wang et al. [10].

## Abbreviations

AEZ: Agro-ecological zone; BL: Billion litres; CARB: California Air Resources Board; CCLUB: Carbon Calculator for Land Use Change from Biofuels Production; CGE: Computable General Equilibrium; COLE: Carbon Online Estimator; CRP: Conservation reserve program; DGS: Distillers grains solubles; EPA: U.S. Environmental Protection Agency; FPF: Forest proration factor; GHG: Greenhouse gas; GREET: Greenhouse gases regulated emissions, and energy use in transportation; GTAP: Global Trade Analysis Project; HWP: Harvested wood product; LCA: Life cycle analysis; LUC: Land-use change; NCASI: National Council for Air and Stream Improvement Inc; RFS2: Renewable fuel standard; SCSOC: Surrogate CENTURY soil organic carbon dynamics submodel; SOC: Soil organic carbon; USDA: U.S. Department of Agriculture; YFS: Young forest shrub.

## Competing interests

The authors declare that they have no competing interests.

## Authors’ contributions

JBD conducted the analysis and writing for this paper with substantial collaboration with SM. MQW and HK also authored and reviewed this paper. All authors read and approved the final manuscript.

## Authors’ information

HK conducted this research while at the University of Illinois at Urbana Champaign. Recently, he has joined the staff at the International Food Policy Research Institute.
